# Bilateral Occipital Lobe Hemorrhages Presenting as Denial of Blindness in Posterior Reversible Encephalopathy Syndrome– A Rare Combination of Anton Syndrome and Encephalopathy

**DOI:** 10.7759/cureus.1746

**Published:** 2017-10-04

**Authors:** Raja Godasi, Chintan Rupareliya, Pradeep C Bollu

**Affiliations:** 1 Department of Neurology, University of Missouri, Columbia, Missouri

**Keywords:** pres, anton syndrome, denial of blindness, occipital hemorrhage

## Abstract

Posterior reversible encephalopathy syndrome (PRES) or reversible posterior leukoencephalopathy (RPL) is an acute neurological syndrome characterized by the development of radiological abnormalities on brain imaging along with clinical manifestations, such as a headache, seizures, encephalopathy, etc. We report the case of a middle-aged male who presented to the emergency department after he woke up with complete blindness and was found to have hemorrhagic PRES. Intracranial hemorrhages were seen in around 15% of patients who presented with this condition. In this article, we review the different types of hemorrhages seen in the setting of PRES and their associations.

## Introduction

PRES was first described in 1992 by Schwartz [1] and was well characterized as a syndrome in 1996 by Hinchey, et al. [2]. It has been described as a clinicoradiological syndrome presenting with clinical features, such as a headache, seizures, encephalopathy, etc., along with cerebral imaging changes most commonly in the posterior white matter [3-4]. The incidence of PRES remains undocumented. The available epidemiological data comes from retrospective studies. It is most commonly seen in middle-aged adults with a mean age ranging from 39 to 47 years. However, cases have been reported in patients with ages ranging from four to 90 years, and a marked female predominance has been noted [4]. The pathogenesis of PRES is unclear, but disturbances in the cerebral blood flow autoregulation, along with endothelial dysfunction, causing vasogenic edema have been postulated as a probable cause [3]. PRES has been associated with a number of conditions, with hypertension being the most common one, while others include eclampsia, immunosuppressive medication, and renal failure [1, 3].

## Case presentation

**Figure 1:**
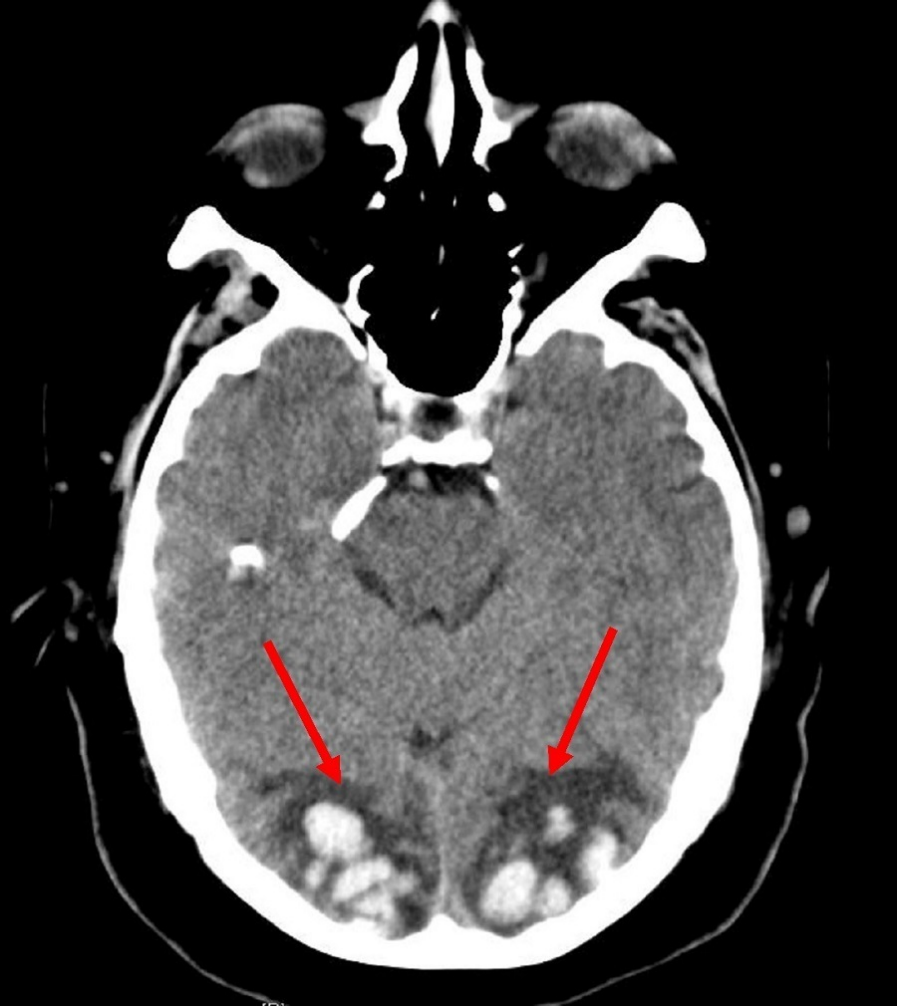
Axial computerized tomography scan of the head showing a hyperintense signal in the bilateral occipital lobes (red arrows)

A 55-year-old white male was brought to the emergency department after he woke up in the morning with complete blindness. He was unable to appreciate any light in both eyes. He also reported a moderate headache after he woke up that morning. Initial examination revealed elevated blood pressure with the systolic blood pressure more than 200 mm of Hg. The patient has a history of hypertension and has been noncompliant with his antihypertensive medications. Neurological examination revealed complete blindness in both eyes and 5 mm wide pupils that were reacting to light equally to direct and consensual light stimulation. The fundoscopy did not reveal any optic disc edema or retinal hemorrhages. Surprisingly, he was not distressed by his profound blindness and even denied his loss of vision during his examination, occasionally confabulating about things he was seeing during visual field testing. His general somatic sensations revealed no focal deficits, except for a mild loss of vibration sense in both lower extremities. There were no gross motor or cerebellar deficits. A preliminary computerized tomography (CT) scan of his head revealed hyperintense lesions bilaterally in the occipital lobes, consistent with hemorrhage as shown in Figure 1. There was no subarachnoid extension nor any intraventricular extension. 

**Figure 2:**
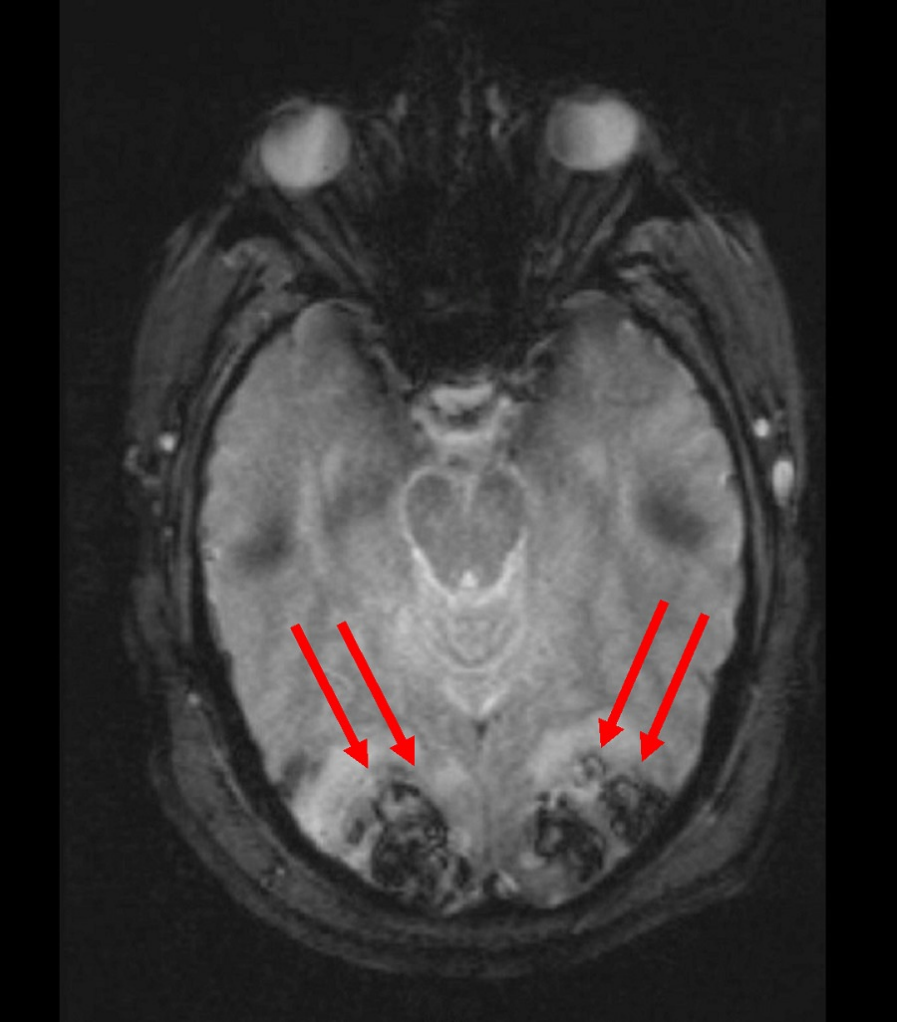
Magnetic resonance imaging of the head with gradient echo sequence showing bilateral occipital hemorrhages (two red arrows)

His preliminary lab results, including the coagulation profile, did not reveal any abnormalities. The patient was admitted to the intensive care unit and his blood pressure was monitored with an intra-arterial line, which revealed even higher blood pressures (systolic blood pressure exceeding 250 mm Hg). Magnetic resonance imaging (MRI) of his brain with gradient echo sequences revealed bilateral occipital lobe hemorrhages without any intraventricular or subarachnoid extension as shown in Figure 2.

There was no associated acute infarction. He had a magnetic resonance angiogram (MRA) which did not reveal any occlusion in his posterior circulation. A subsequently done magnetic resonance venogram did not reveal any venous thrombosis. His blood pressure was initially controlled with intravenous labetalol and later switched to a nicardipine drip. He had a prolonged hospital course with improvement in his vision at the time of his discharge. An   Institutional Review Board (IRB) approval from the University of Missouri was obtained for the publication of this case report. The IRB approval number for this case is 230320.

## Discussion

**Figure 3:**
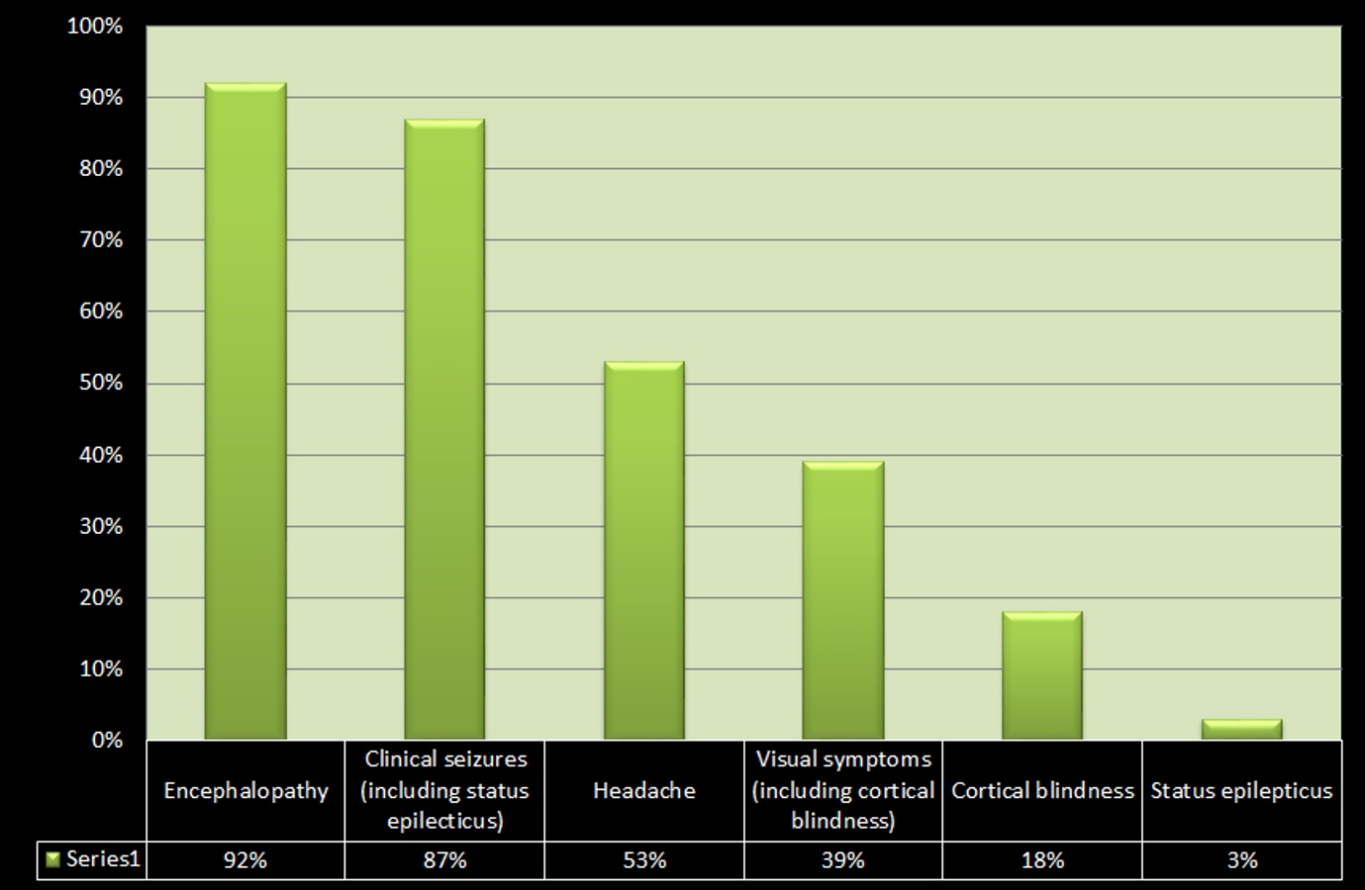
Bar chart demonstrating the frequency of various clinical manifestations in posterior reversible encephalopathy syndrome

Posterior reversible encephalopathy syndrome (PRES) is characterized by a variety of symptoms ranging from a headache, altered mental status, and visual loss to seizures and loss of consciousness, accompanied by a typical CT or MR imaging pattern showing diffuse, symmetric edema predominantly affecting white matter, particularly in the posterior parietal and occipital lobes. Asymmetric involvement or involvement of other areas of the brain may also be seen in some cases [1, 3, 5]. The frequency of various clinical manifestations in PRES, according to a retrospective cohort study by Lee, et al., is demonstrated in Figure 3 below [5].

Our patient demonstrated complete blindness in both eyes, along with a denial of blindness (visual anosognosia), which, in the setting of hyperintense lesions in the bilateral occipital lobes on CT scan, can be attributed to cortical blindness. This symptomatology is called Anton syndrome (also called Anton-Babinski syndrome). Complete loss of vision due to cortical blindness is an uncommon symptom of PRES, which is seen only in about 18% of PRES cases, according to one study [5].

PRES has been noted to be associated with a number of conditions, including hypertension, immunosuppressive medications, renal failure, eclampsia, chemotherapy agents, and autoimmune diseases, such as systemic lupus erythematosus (SLE) [1, 3, 6]. Among these, hypertension appears to be the most common comorbidity [5]. The pathogenesis of PRES is thought to be related to endothelial cell dysfunction/injury leading to blood-brain barrier leakage with resultant cortical and subcortical vasogenic edema [2, 7].

**Figure 4:**
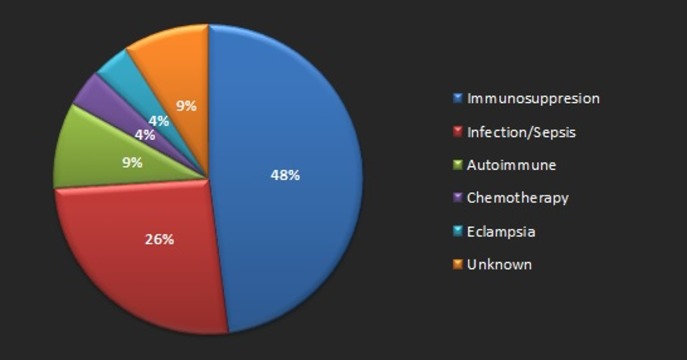
Pie chart demonstrating various clinical associations with hemorrhagic posterior reversible encephalopathy syndrome

The diagnosis of PRES is done with a combination of history and physical examination followed by neuroimaging. Neuroimaging is an indispensable tool in the diagnosis of PRES. MRI has been demonstrated to be superior to CT imaging, which tends to show normal findings or non-specific abnormalities in some cases. Hence, MRI should always be obtained in suspected cases of PRES, even if CT was done and shows normal imaging findings [4]. Our patient had hyperintense lesions in the bilateral occipital areas on CT scan, which were corroborated by an MRI scan that was done later on. Studies have demonstrated that the incidence of parenchymal or sulcal subarachnoid hemorrhage in PRES is around 5-17% of the patients with PRES [6, 8-9]. Doss-Esper, et al. have proposed that the mechanism of hemorrhage in PRES could be due to either postischemic reperfusion injury (leading to multifocal brain hemorrhages or severe hypertension) or impaired autoregulation (leading to rupture of pial vessels leading to nonaneurysmal subarachnoid (sulcal) hemorrhage) [10]. Immunosuppression was the most frequently identified comorbidity in patients with hemorrhagic PRES [8]. Figure 4 shows the percentage distribution of comorbidities associated with hemorrhagic PRES, according to a study done by Hefzy, et al. [8].

Our patient did not have any of the known clinical associations that were associated with hemorrhage in the setting of PRES. This point distinguishes our case from others, as most cases of hemorrhagic PRES have at least one of the comorbidities described above with only 9% of the total cases having no known clinical associations. Blood pressure has not been shown to have an effect on the development of hemorrhage in PRES as the incidence rate of hemorrhage was similar in normotensive PRES cases, as well as those with moderate to severe hypertension [8]. Once PRES has been diagnosed, treatment should be started promptly; this includes the correction of underlying causes by means of blood pressure control, withdrawal of toxic agents, such as immunosuppressive medications, dialysis, etc. [4]. Control of high blood pressure is an important part of the management of PRES, and the aim is to decrease the mean arterial blood pressure by 20-25% within the first two hours and less than 160/100 mmHg within the initial six hours. Intravenous (IV) antihypertensive agents, such as labetalol and nicardipine, may be used to help with blood pressure regulation [4]. Seizures, when present, can be controlled by means of anti-epileptic medications [4-5]. However, long-term antiepileptic treatment is not indicated [5]. Our patients did not have any associated conditions, other than hypertension, and hence was treated with antihypertensive medications.

## Conclusions

PRES is an acute neurological condition presenting with the wide range of symptoms, including headaches, seizures, and altered mental status. Hemorrhage in the setting of PRES is uncommon. The syndrome usually has a good prognosis. Our case had a rare presentation of hemorrhagic PRES in the setting of poorly controlled blood pressure and visual anosognosia.
